# Supplementary material for: The interactome of intact mitochondria by cross-linking mass spectrometry provides evidence for coexisting respiratory supercomplexes
Source: Mol Cell Proteomics. 2017 Dec 8;17(2):216–32. doi: 10.1074/mcp.RA117.000470 (PMC5795388; doi:10.1074/mcp.RA117.000470)
Supplement: Supplemental Data [file supp_RA117.000470_133922_0_supp_23978_fzffwf.zip › spectra_annotation/mito_spectra_annotation/283-1-25-1-18-1.pdf]

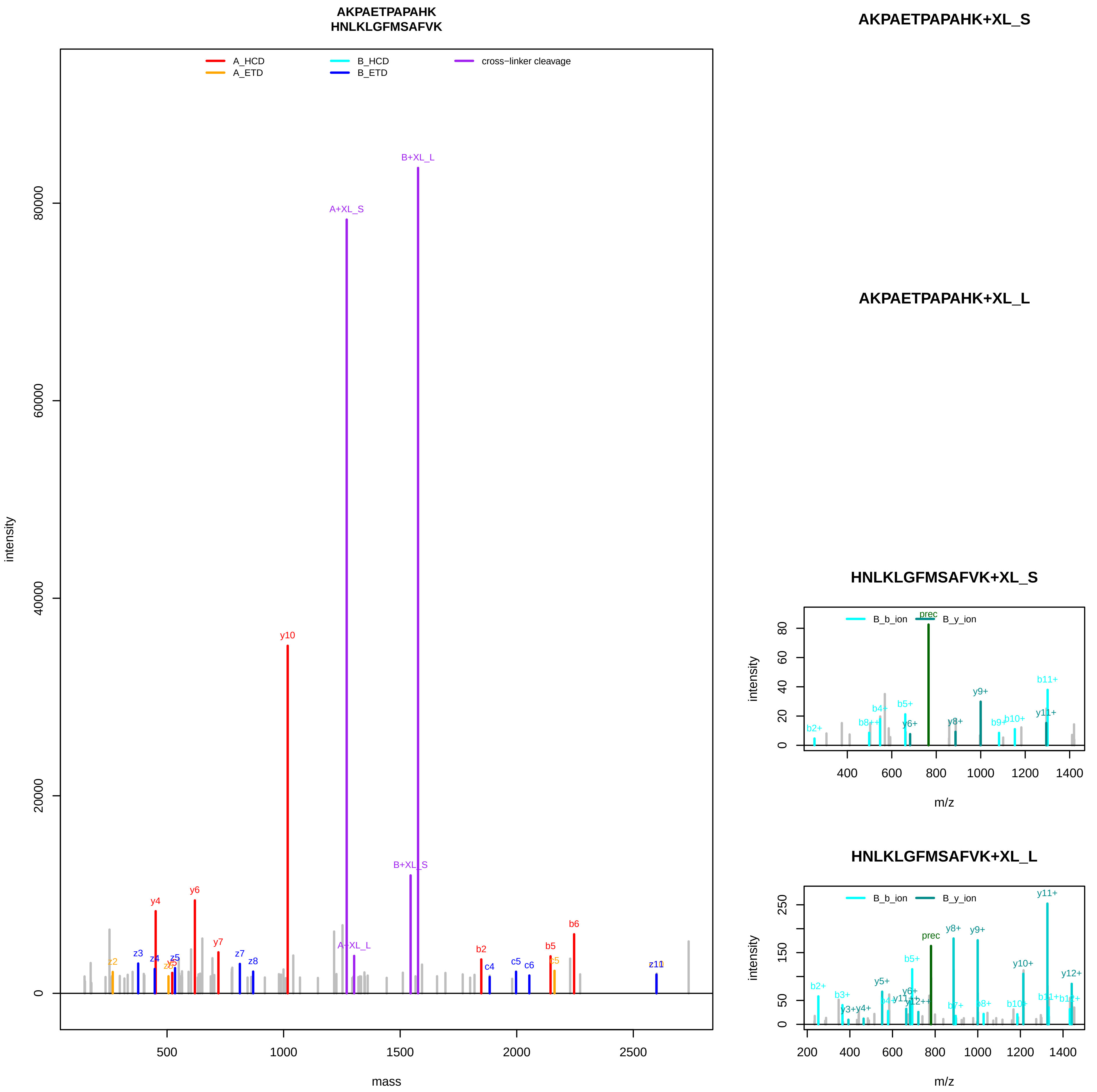
AKPAETPAPAHK
HNLKLGFMSAFVK
A_HCD
A_ETD
B_HCD
B_ETD
cross−linker cleavage
intensity
mass
0
20000
40000
60000
80000
500
1000
1500
2000
2500
A+XL_S
B+XL_L
B+XL_S
A+XL_L
y4
y6
y7
y10
b2
b5
b6
z2
z3
z4
z5
z7
z8
z11
c4
c5
c6
AKPAETPAPAHK+XL_S
AKPAETPAPAHK+XL_L
HNLKLGFMSAFVK+XL_S
B_b_ion
B_y_ion
prec
m/z
b2+
b4+
b5+
b8++
y6+
y8+
y9+
b9+
b10+
b11+
y11+
HNLKLGFMSAFVK+XL_L
b3+
y3+
y4+
y5+
y8+
y10+
y11+
y12+
b12+
